# PathogenMip Assay: A Multiplex Pathogen Detection Assay

**DOI:** 10.1371/journal.pone.0000223

**Published:** 2007-02-21

**Authors:** Michael S. Akhras, Sreedevi Thiyagarajan, Andrea C. Villablanca, Ronald W. Davis, Pål Nyrén, Nader Pourmand

**Affiliations:** 1 Stanford Genome Technology Center, Stanford University, Palo Alto, California, United States of America; 2 Department of Biotechnology, Royal Institute of Technology, Stockholm, Sweden; National Institute for Communicable Diseases, South Africa

## Abstract

The Molecular Inversion Probe (MIP) assay has been previously applied to a large-scale human SNP detection. Here we describe the PathogenMip Assay, a complete protocol for probe production and applied approaches to pathogen detection. We have demonstrated the utility of this assay with an initial set of 24 probes targeting the most clinically relevant HPV genotypes associated with cervical cancer progression. Probe construction was based on a novel, cost-effective, ligase-based protocol. The assay was validated by performing pyrosequencing and Microarray chip detection in parallel experiments. HPV plasmids were used to validate sensitivity and selectivity of the assay. In addition, 20 genomic DNA extracts from primary tumors were genotyped with the PathogenMip Assay results and were in 100% agreement with conventional sequencing using an L1-based HPV genotyping protocol. The PathogenMip Assay is a widely accessible protocol for producing and using highly discriminating probes, with experimentally validated results in pathogen genotyping, which could potentially be applied to the detection and characterization of any microbe.

## Introduction

Multiplex assays to identify the presence of a wide variety of microbes within a specimen will have a profound impact on the efficient management of disease treatments and prevention, clinical follow-up studies, and the development of new therapies of prophylactic or therapeutic vaccines. Here we describe a complete protocol for a multiplex assay in single-tube reactions useful in pathogen diagnostics. We compare our results to those obtained from a conventional general-primer based amplification [1] approach when genotyping primary tumor genomic DNA extracts from cervical cancers.

Nucleic-acid based diagnostic techniques typically rely on unbiased PCR amplification [2] of the targets, which requires a set of general PCR amplification primers to hybridize upstream and downstream of the variable region, followed by either real-time PCR [3], hybridization assays [4] or template sequencing [5] for read-out interpretation. Multiplex PCR [6], has the potential to decrease cost, time and effort in pathogen diagnostics [7]. Engineering an efficient multiplex PCR requires laborious strategic planning in primer design, nucleotide concentrations, optimal salt and buffer conditions, and use of chemical adjuvants [8] and is rarely capable of achieving multiplex degrees greater than a 20-plex.

The field of molecular diagnostics is experiencing a revolution in regards to theranostics, the integration of diagnostic technologies with therapeutic applications [9]. As the number of biomarkers for microbial agents and disease markers [10] increases, simultaneous detection of multiple agents or risk factors implicated in particular clinical syndromes or diseases that share similar epidemiological features will become highly desirable. Groundbreaking research has been performed with the advent of a ligase-mediated gene detection technique [11] and the many technologies based on the work of Landegren *et al* [12]. Ligase-based technology has led to myriad promising techniques such as the padlock probe [13], [14], and the proximity ligation assay [15], which have both been investigated as potential pathogen diagnostic methods [15], [16]. Molecular Inversion Probe (MIP) technology [17], has contributed new features to padlock probes, resulting in an ultra high-throughput method for SNP detection [18] and further investigation as a potential quantitative method [19].

**Figure 1:**
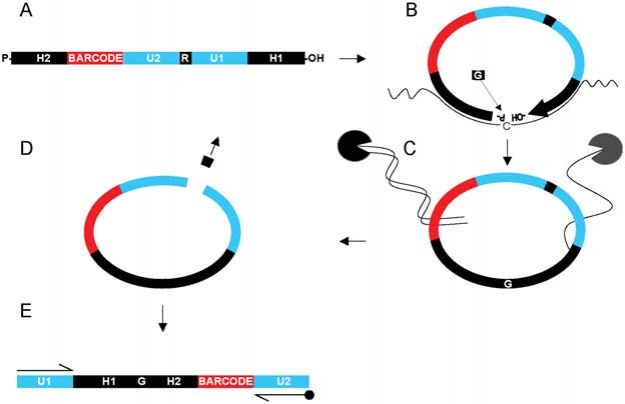
Schematic overviews of molecular inversion probe technology. **A**) Synthetic oligonucleotide containing following four regions; i) H1 and H2: homology regions comprised of unique continuous 40–50 base pair fragments for target recognition ii) BARCODE: molecular barcode comprised of a 20 base pair DNA tag for target identification iii) U1 and U2: universal primer regions for inverted probe amplification, and iv) R: restriction site for probe linearization. **B**) Upon target recognition, a DNA polymerase fills the missing gap in between the juxtaposition of the probes' flanking ends, and through the activity of a DNA ligase the probe is circularized. In all cases the missing nucleotide is a “G”. **C**) Circular DNA enrichment through DNA degradation by enzymes Exonuclease I and III. **D**) Probe linearization restriction site cleavage. **E**) All reacted and inverted probes are amplified with universal primers, of which one is biotinylated for subsequent amplicon validation.

As a model assay for MIP pathogen diagnostics, we chose to target the Human Papillomavirus (HPV), which is well known for its cancer-associated cervical infections and for the existence of multiple genotypes [20]. Based on the oncogenic potential, the subtypes are classified as “high-risk” or “low-risk” HPVs. We developed a 24-plex PathogenMip assay to target HPV genotypes commonly associated with cervical cancer progressions [21]. The MIP assay was performed in four sequential enzymatic driven reactions (Figure 1). Target recognition sites for the MIPs in this assay were designed using a previously described software tool, PathogenMIPer [22]. We also attempted to introduce a novel ligase-dependent probe production at equal or increased assay effectiveness, which would be less expensive than direct manufacture of full-length probes. Pyrosequencing [23] and hybridization [24] were applied to the HPV amplified L1-region to validate the results obtained from the PathogenMip Assay.

## Results

### Ligation-based probe construction

**Figure 2:**
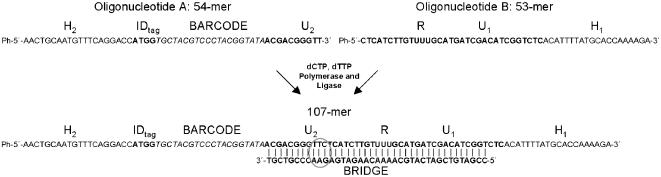
Ligase-based probe construction for the PathogenMip Assay. Seen in the figure is the sequence for probe-16 prior to inversion. Two shorter fragments were synthesized and hybridized to a bridge complementary to the universal primer regions, common for all probes used in the assay. The coupling of the shorter fragments was mediated by polymerase and ligase activity to achieve maximum yield.

For cost-efficient production of MIPs, two shorter DNA constructs (denoted **A** and **B**) were produced in-house and ligated through a ligation-based production assay (Figure 2). Several different assay protocols were examined, in which: i) dCTP, dTTP and a polymerase (Stoffel fragment) were added to the ligation reaction, ii) bridge concentrations were varied from ratios of 2∶1 down to 1∶4 with respect to the concentrations of the **A** and **B** constructs, and iii) Ampligase was replaced with a T4 DNA ligase based kit (Fast-Link DNA-ligation kit, Epicenter Biotechnologies; Madison, WI). Gel-electrophoresis was used as read-out for all optimizations (data not shown).

Thus, best results with regards to yield and purity were achieved by inclusion of Stoffel fragment, dCTP and dTTP, and use of equal concentrations of bridge, **A**, and **B** constructs. No noticeable differences were seen with the use of different ligase-enzymes, and Ampligase was used in subsequent tests. Using this protocol, the ligation step produced clear DNA gel-bands at a 100 base pairs, which were isolated and purified. All 25 probes (including the reference probe, rMIP, which targets the β-globin region in human DNA) were pooled together into a mixture of roughly equal probe concentrations with a total concentration of 5 fmol/probe.

### PathogenMip Assay validation

Our primary goal was to specifically genotype each target while using a complete 25-plex MIP mixture (24 HPV probes plus the control rMIP), without generation of false-positives or negatives. To enhance sensitivity and selectivity, we explored a variety of initial probe concentrations and thermoprofiles for the initial ligation and gap-filling step (Figure 1B). All optimizations of the PathogenMip Assay were based on UV-visualization of DNA band intensities after size separation by gel electrophoresis (data not shown). The initial probe concentrations yielded an appropriate signal when using 5 fmol of each contributing probe. Cycle times and annealing temperatures in the initial reaction step yielded an efficient protocol, comprising 5 cycles of denaturing and two annealing times at 50°C and 37°C.

Using the optimized conditions, all 21 HPV plasmids and 3 oligonucleotide targets (see Materials and Methods, *DNA materials*) were detected with the PathogenMip Assay, and sequencing individually validated each result. The positive control, rMIP, was successfully validated through a reaction with a commercially available human genomic DNA, without positive matches to any of the HPV genotypes.

### PathogenMip Assay performance

To further investigate assay sensitivity, we used a fixed concentration of the MIP mixture at 5 fmol/probe in the ligation step, while varying the amount of target DNA. This experiment was performed in two versions. The first set of reactions contained pure plasmids. The second set of reactions contained, in addition to the target plasmids, a constant amount (200 ng) of non-reactive, commercially available human genomic DNA. By spiking with human genomic DNA we hoped to approximate more realistic sample environments for which the assay was constructed. So that the experiments would be of significant value, the probe mixture did not include the rMIP, since rMIP would react with the human genomic DNA and give false quantification data. The plasmid concentrations ranged from 100 ng to 10 fg through a 10-fold dilution series. The original detection limit goal for the assays was 1 pg in the pure plasmid environment and 1 ng for plasmid in the presence of 200 ng human genomic DNA (data not shown), when cycling the PCR reaction at 35 cycles. By using an alternative PCR protocol, working at a higher annealing temperature (58°C) for 60 cycles, we were able to improve the sensitivity of the assay. No background was observed with either of the PCR conditions, but a 10-fold higher sensitivity was obtained with the more extensive 60 cycle-protocol. The new detection limit values were 100 fg for the pure plasmid and 100 pg for plasmid in the presence of 200 ng human genomic DNA (data not shown). Targets in all reactions were correctly typed as judged by L1-pyrosequencing validation.

**Figure 3:**
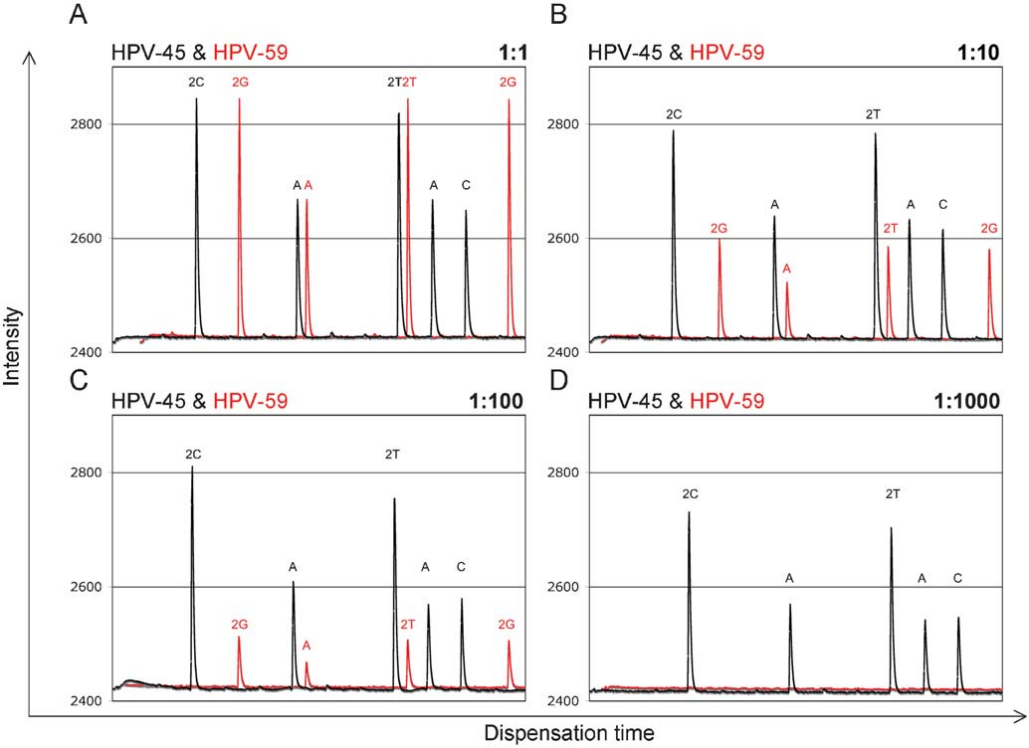
Probe selectivity. The figure depicts superimposed pyrosequencing (dispensation time vs. light signal intensity) diagrams for two probes following the MIP reactions, targeting HPV-45 (black) and HPV-59 (red), mimicking a double HPV infection. **A**) The initial DNA amount of each contributing plasmid was 100 ng and equal levels of sequencing intensities are seen. **B**) The DNA amount of HPV-45 plasmid remained at 100 ng, while HPV-59 plasmid was set at 10 ng resulting in ∼2-fold lower signal intensity. **C**) DNA amounts were set at 100 ng for HPV-45 plasmid and 1 nanogram for HPV-59 plasmid, which was observed as a ∼3-fold decrease in signal intensity. **D**) DNA amounts of 100 ng of HPV-45 plasmid and 100 pg of HPV-59 plasmid resulted in signal from HPV-45-probe without a measurable signal from the probe for HPV-59.

In multiple HPV-infections, the viral load of each contributing genotype can differ by orders of magnitude [5]. To obtain a deeper understanding of the assay's capabilities when faced with such challenges, we set up a series of reactions to mimic this possibility. Here we chose a two-plasmid detection model in which plasmid HPV-45 was present at a fixed amount of 100 ng, while the second plasmid HPV-59 was present in a wide concentration range. In this scenario, we were able to obtain interpretable data for both genotypes for up to 2-logs difference in target DNA concentration, using the PathogenMip Assay on the different in parallel with pyrosequencing to confirmresults (Figure 3).

### Genotyping of primary tumor genomic DNA extracts

**Figure 4:**
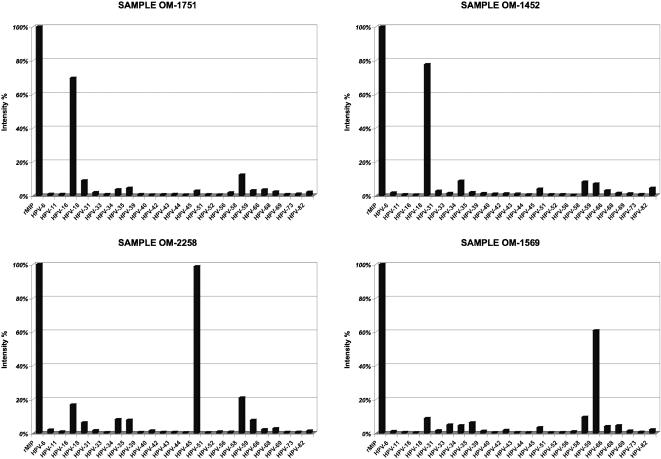
Bar-histograms representing fluorescence intensities from the in-house barcode-chip for genotyping genomic DNA extracts from tumor samples derived from four patients with cervical cancers. Seen in the figure are four examples of single HPV infections, one from each genotype, observed in the sample set. HPV-16 was genotyped in sample OM-1751, HPV-18 in OM-1452, HPV-45 in OM-2258 and HPV-59 in OM-1569. The signal-intensities were normalized to the intensity of the peak for the reference probe targeting human β-globin gene (rMIP). The remaining bars constitute of the reaction background signal.

The ultimate objective was to screen samples with unknown viral load. A total of 20 commercially available primary tumor genomic DNA extracts were collected from patients with cervical cancers. The DNA samples were screened through the HPV PathogenMip Assay and detected using both methods (pyrosequencing and the in-house barcode-chip assay). The results were validated by genotyping the samples in parallel using the conventional general primer based nested PGMY09/11 and GP5+/6+ amplification protocol. For all tumor-drived samples, the agreement among the methods (PathogenMip Assay and conventional genotyping) was 100%, with 10 samples positive for HPV-16, 5 for HPV-18, two for HPV-45, two for HPV-59 and one sample returning as HPV-negative. When using 200 ng of genomic DNA extract for the PathogenMip assay, equal levels of detection efficiency were obtained for both methods (Figure 4).

### Multiplexing capabilities

**Figure 5:**
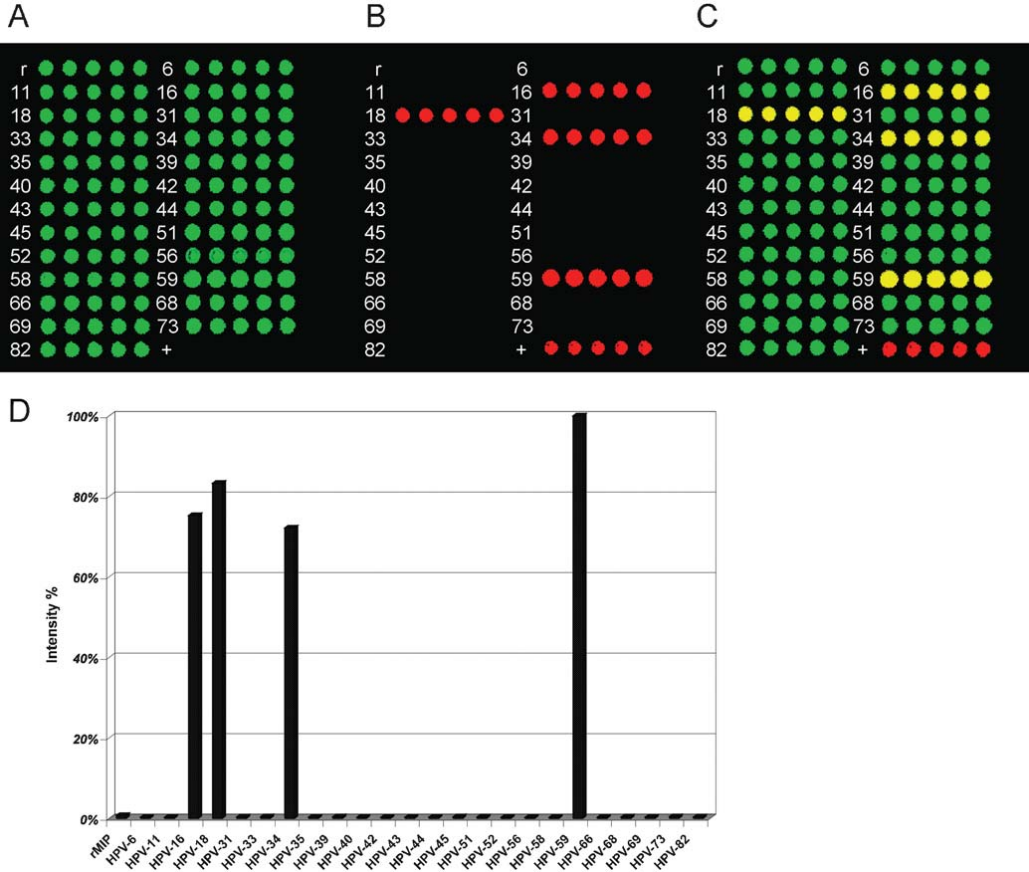
Results from the in-house barcode-chip screening of a quadruple HPV-infection. A) In-house barcode-chip at wavelength 635 emitting the fluorophore signal for internal control Linker A, positive for all oligonucleotides in the chip. B) Wavelength 535 emitting the fluorophore signal for the biotinylated MIP amplicons and the positive control. The in-house barcode-chip was positive for all four HPV genotypes in the pool (HPV-16, -18, -34 and -59). C) Wavelength ratio 535/635 representing the signal ratio from the positive samples to that from linker A. D) Bar histograms representing mean value signal intensities for 2 arrays analyzed with 5 replicates per chip. The highest peak signal for HPV-59 was used for normalization for all the values in the graph for visual purposes. The variability in probes intensities can be explained by individual differences in probe composition and a batch-to-batch variation in the production of the probes.

We also performed the PathogenMip assay screening a mixture of multiple HPV plasmids set at equal concentrations of a 100 ng. Many patients have multiple HPV infections and, though rare, such patients are known to carry as many as five subtypes [5], so we set as our goal to prove that it is feasible to detect four simultaneous infections at equal viral loads using the PathogenMip Assay. HPV plasmids of four high-risk HPV genotypes (-16, -18, -34, and -59) were pooled in equal amounts at a 100 ng/plasmid, and screened simultaneously in triplicate using the complete 25-plex-probe mixture. The reactions were then validated by both pyrosequencing and hybridizations methods. All four HPV subtypes were detected at similar detection intensities differing by ±20% from the median value, when hybridized through the barcode to an in-house barcode-chip (Figure 5).

## Discussion

A complete pathogen detection assay based on MIP technology is under construction. Here, we describe a successful model assay targeting 24 HPV genotypes most commonly associated with the progression to cervical cancer. Our pilot subset can now be extended to include all known sequenced HPV genotypes, as multiplexing up to 20,000 using the MIP method for human SNP genotyping, has been demonstrate to be feasible (www.affymetrix.com/technology/mip_technology.affx), and this would more than cover the existing variability in the HPV genome.

In the case of a double HPV infection, the fact that the PathogenMip Assay detected and distinguished between two contributing viral loads that differed by 2-logs at an input level of 200ng genomic DNA is quite promising. While DNA yields from tumors vary widely, this level is in the mid-range of recovery in our experience. Exploration of a more full matrix of DNA double-loads and inputs is under way.

Primary tumor genomic DNA extracts were efficiently genotyped for a wide dynamic range of viral loads (Figure 4). The reference probe (rMIP) provided a positive reference control in each performed reaction (Figure 4) and, if used to normalize viral loads against total human genomic DNA content, may allow the researcher a tool to compare viral loads from genomic DNA extracts. The results of the PathogenMip Assay genotyping were in 100% agreement with the conventional genotyping methods.

Our goal was to provide a complete, experimentally validated assay in a ready-to-use format, which could be applied generically to any pathogen based on genome target recognition. PathogenMiper [22] serves as an *in silico* multiplex test system, whereby one can check the feasibility of designing a MIP assay for the specific targets one might want to include in a multiplex detection assay.

Microbial genomes commonly incur variations from insertions, deletions, or mutations that can generate false-negative results if the variation resides within a probes' targeted region. A more complete diagnostic approach would be to include multiple probes per genotype; this would also provide deeper epidemiological insights in functional genome patterns and variations, i.e. mutations coding for drug resistance [25]. Conventional PCR-based genotyping methods are restricted to reside within conserved genomic regions, which limits the choice of genotyping targets [1], [26]. These methods are not robust to genomic changes, a problem that can be at least partially addressed by the multiplexing possible in the PathogenMip Assay.

In the current incarnation of the assay, we have incorporated a ligation step to facilitate probe generation. An oligonucleotide complementary to the universal primer region, which is present in each probe, can be used as a bridge so that one longer probe can be ligated together from two smaller constructs, and length requirements for probe synthesis are therefore cut in half. Longer oligonucleotides result in higher initial costs, and have been known to compromise sequence quality [27]. With our new ligation-based probe production, we introduce cost-efficient probe production with possibilities for innovation and potential increase in efficiency. Probe production using the ligation method can easily be performed in any academic or other research institution with budget constraints, using only standard labware and avoiding core facility based research. We are currently investigating parameters for increased ligation efficiency and probe production. The goal of such efforts would be a more homogeneous distribution of probe concentrations, thus enabling one to perform high-throughput production via one-tube reactions and use of fully automated workstations, e.g. Biomek FX (Beckman Coulter, Palo Alto, CA). Parameters that could be varied to this end include ligation at higher temperatures (preferred 65°C) to allow improved incubation profiles, and higher cycle numbers [28]. Use of modified nucleotides, e.g. locked nucleic acids (LNA) [29] in the bridge would allow for higher melting temperatures (T_m_) for the universal primer regions. Also by replacing the conventional gel-purification protocol with one based on single-strand DNA separation, higher purity of ligated versus non-ligated DNA can be achieved, resulting in a more sensitive assay (data not shown). The introduction of a second universal primer region would require a ligation-based production assay based on three contributing constructs and two bridges complementary to the two universal primer regions. By designing the new probes so that the two primer regions flank the barcode region, a three-way ligation currently under investigation would result in new design features regarding assay possibilities and efficiency.

Although our preferred readout for the PathogenMip Assay uses sequencing or hybridization of the identifying barcode, we also used sequencing of the target-homologous regions of the PathogenMip inverted probes to double check target identity. In addition, we performed conventional HPV identification through the sequencing of the L1 regions. All three assays (PathogenMip/barcode, PathogenMip/target region, and HPV L1 sequencing) produced the same final result with respect to target identification, regardless of whether the readout was performed by pyrosequencing or hybridization methods. Pyrosequencing of the ID-tag was more time and cost-efficient than either hybridization to the in-house barcode-chip or the L1-hybridization-assay. Multiple-primer pyrosequencing, however, is limited to reactions of 20-plex or lower. A higher degree of multiplexing in a sequencing-based approach might be achieved through use of a high-throughput configuration of the pyrosequencing method; for example, the one offered by 454 Life Sciences (http://www.454.com/applications/index.asp). However, when high capacity is required, hybridization-based methods show more potential than sequencing methods, and here the PathogenMip Assay has the advantage of providing more discrimination through its targeted probes than would a standard hybridization to highly conserved regions. The use of ID-tag markers incorporated in the probes simplified sequence read-out in the current PathogenMip Assay, but a more forward looking approach involves refinement of the in-house barcode-chip hybridization piloted here. We anticipate that a high-throughput configuration of the current PathogenMip assay will be achievable through use of the commercially available Affymetrix TAG4-array [30], which is based on detection of barcodes through hybridization similar to the process described here. The TAG4-array covers a total of 80K features, which includes a total of 16,000 unique barcodes in five replicates.

Sensitivity levels obtained with the current assay were sufficient for a functional assay and were improved with the use of a more extensive amplification protocol. Previously described ligation-based genotyping methods [16], [31] have shown sensitivity ranging down to nanograms of genomic DNA for accurate genotyping. These methods have placed careful emphasis on the amplification step by either rolling circle amplification method [32] or stringent reaction cleanup with reversible magnetic biotin-streptavidin separation [33], followed by design of a PCR run at high annealing temperatures and high cycle numbers, which also could be performed with the use of LNA universal primers [34]. Further consideration of these parameters should allow improvement in sensitivity of a next-generation PathogenMip Assay.

The potential uses for an assay that simultaneously detects multiple organisms based on differences in DNA sequences are almost unlimited. MIP-based diagnostics have already progressed in the field of human SNP-detection and cancer diagnostics [10] with ongoing clinical trials. Microarray techniques have gained ground in the pathogen diagnostic arena, with various genotyping chips such as the HPV-assay PapilloCheck (www.greinerbioone.com) upcoming. By combining the benefits of a high-throughput multiplex MIP-assay prior to PCR amplification and of a post-PCR hybridization based chip read-out, researchers can expand the scope of their surveillance to reach new insights into epidemiology and clinical management. We therefore encourage researchers with expertise in various pathogenic microbes to adapt the PathogenMip Assay to accomplish similar detection screenings as performed in this study. By combining the different models, we can reach a common goal toward a standardized virus-, bacteria-, or fungi- chip for routine diagnostics and epidemiological studies.

## Materials and Methods

### Experimental design

**Figure 6:**
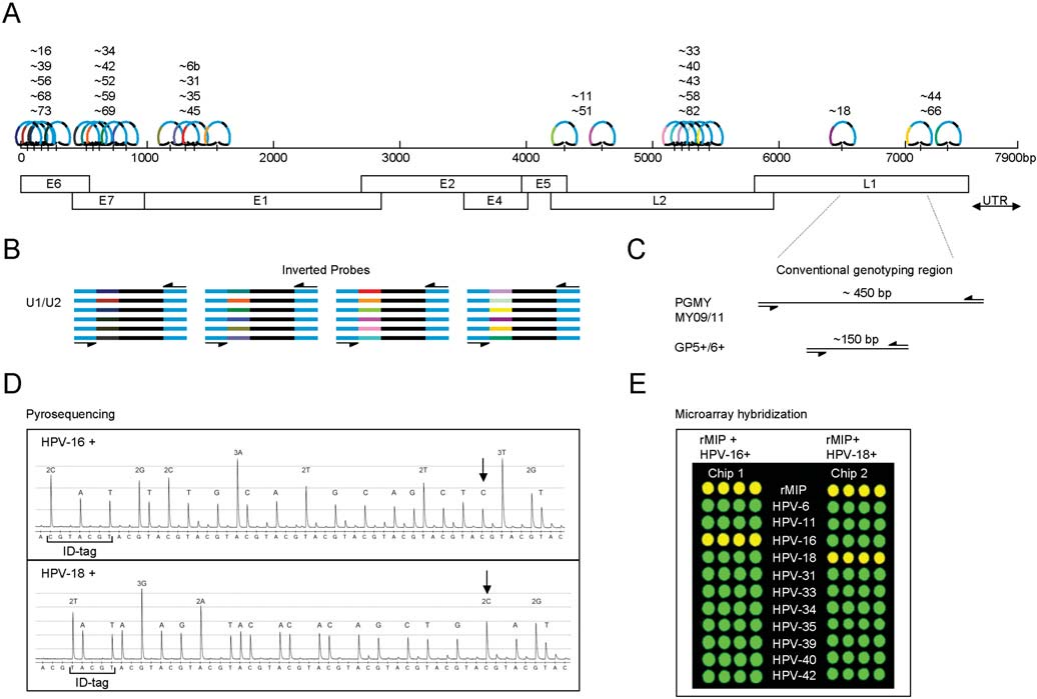
Schematic overview of the PathogenMip Assay. **A**) The 24 probes included in the assay are situated at their respective target sites on the approximately 8000 base pairs of double stranded HPV genomic DNA. Early genes (denoted E) code for virus integration and replication and late genes (denoted L) encode the viral capsule creation. The probes recognize ∼40 base pair fragments unique for each targeted genotype. **B**) Following enzymatic inversion of reacted probes and universal amplification, the amplicons are used for subsequent appropriate HPV genotype screening. **C**) Conventional HPV genotyping takes a different approach, in which the nested primer pairs PGMY09/11 and GP5+/6+ amplify respectively ∼450 base pair and ∼150 base pair fragments that, through an appropriate readout process, will make up the basis for genotyping. These primers are restricted to the highly conserved genomic regions, most commonly found in the L1 gene. **D**) Multiple-primer DNA Pyrosequencing of an incorporated ID-tag. The diagrams depict the complementary sequence of the investigated probes -16 and -18. Marked in the figure is the ID-tag for each probe and the point of ligation where the probes circularized, incorporation of a dGTP, seen here as the complementary “C”. **E**) The in-house barcode chips here used to detect one HPV-16 positive, and one HPV-18 positive in human genomic DNA presence as seen with a positive rMIP in both chips.

Our goal was to design an assay to simultaneously distinguishing among over 100 HPV genotypes that differ only slightly in their genomic sequences (up to 90% homology). The critical regions of the probes that recognize and distinguish among the HPV subtypes were generated using PathogenMIPer, a previously described software tool [22]. Each target sequence was designed through multiple, successive steps of evaluation of candidate sequences, based on user-defined criteria, followed by a BLAST search against non-target genomes potentially present in the sample that could otherwise cause background noise or interfere with signal. For the study, we included 24 HPV genotypes (Table 1 and Figure 6A) most commonly associated with cervical cancer progression [20]. Our design strategy allowed the probes to target any region of the approximately 8000 bp viral genome. Probes that targeted the E6/E7 genes were preferred, since these genes are most commonly associated with cervical cancer progression [35]. The HPV-PathogenMip Assay also included a reference probe (rMIP) targeting the β-globin region in human DNA (Table 1). Validation was performed by either ID-tag based multiple-primer DNA sequencing [5] or barcode hybridization, using our in-house microarray chip setup, the in-house barcode-chip. Each ID-tag was carefully designed so that it was easily distinguishable when up to four probes were sequenced in parallel during the validation reaction, without primer cross hybridization (Table 2 and Figure 6B). A conventional genotyping assay was run in parallel, based on nested PCR amplification (Figure 6C). The PCR amplicons were followed by sequencing or hybridization interpretation (Figure 6D–E).

**Table 1:** Probes used in this version of the PathogenMip Assay.

Probe	HPV genotype	Cancer risk group	Accession number	Gene
rMIP	-	-	AY310318	HBB
6b	HPV 6b	Low-risk	X00203	E1
11	HPV 11	Low-risk	M14119	E5
16	HPV 16	High-risk	K02718	E6
18	HPV 18	High-risk	X05015	L1
31	HPV 31	High-risk	J04353	E1
33	HPV 33	High-risk	M12732	L2
34	HPV 34	High-risk	X74476	E7
35	HPV 35	High-risk	M74117	E1
39	HPV 39	High-risk	M38185	E6
40	HPV 40	Unspecified	X74478	L2
42	HPV 42	Low-risk	M73236	E7
43	HPV 43	Low-risk	NC_005349	L2
44	HPV 44	Low-risk	U31788	L1
45	HPV 45	High-risk	X74479	E1
51	HPV 51	High-risk	M62877	L2
52	HPV 52	High-risk	X74481	E7
56	HPV 56	High-risk	X74483	E6
58	HPV 58	High-risk	D90400	L2
59	HPV 59	High-risk	X77858	E7
66	HPV 66	High-risk	U31794	L1
68	HPV 68	High-risk	NC_004710	E6
69	HPV 69	Unspecified	M73258	E7
73	HPV 73	Unspecified	U21941	E6
82	HPV 82	Unspecified	X94165	L2

For each probe, information is provided regarding which HPV genotype (or human gene) it targets, NCBI accession numbers, and the genes where the recognition sites lie.

**Table 2:** ID-tags and multiple-primer mixes of complementary barcode sequencing primers (cBarcodeS) for effective DNA sequencing readout.

	ID-tags			
Multiple Primer Pool	CCAT	TTAT	GGAC	GGACCT
Mix 1	cBarcodeS-16	cBarcodeS-68	cBarcodeS-35	cBarcodeS-51
Mix 2	cBarcodeS-31	cBarcodeS-11	cBarcodeS-59	cBarcodeS-56
Mix 3	cBarcodeS-45	cBarcodeS-52	cBarcodeS-39	cBarcodeS-58
Mix 4	cBarcodeS-66	cBarcodeS-18	cBarcodeS-69	cBarcodeS-34
Mix 5	cBarcodeS-6	cBarcodeS-33	cBarcodeS-42	cBarcodeS-82
Mix 6	cBarcodeS-40	cBarcodeS-43	cBarcodeS-73	cBarcodeS-44
cBarcodeS-rMIP	**CAGGG**			

For verification purposes, the first 5 bases for the rMIP obtained from 2 cyclic (ACGT) dispensations are also included.

### DNA materials

All oligonucleotides used in the assay (Table 3) were produced in-house (Stanford Genome Technology Center). HPV plasmids were kindly provided by Dr. E. M. de Villiers (DKFZ; Heidelberg, Germany) (HPV-6, 11, 16, 18, 40, 45, 51 and 73); HPV-33, 34, 39, 42 and 66 by Dr. M. Favre (Institute Pasteur; Paris, France); HPV-31, 35, 43, 44, and 56 by Dr. A. Lorincz (Digene Corporation; Gaithersburg, MD); HPV-59 and 82 DNA by Dr. T. Matsukura (National Institute of Health; Tokyo, Japan) and HPV-52 by Dr. W. Lancaster (Wayne State University School of Medicine; Detroit, MI). The HPV plasmids were normalized at 100-ng/µl using a ND-100 Spectrophotometer (NanoDrop, Wilmington, DE). Because we lacked plasmids containing HPV subtypes 58, 68, and 69, we manufactured synthetic oligonucleotide targets, denoted as TEMP-58, -68 and -69 to complement the homologous regions of our probes for these genotypes (Table 3B). Twenty commercially available primary tumor genomic DNA extracts were obtained from Oncomatrix, Inc. (www.oncomatrix.com). Commercially available human genomic DNA G3041 (Promega, Madison, WI) was also used for assay validation.

**Table 3:**
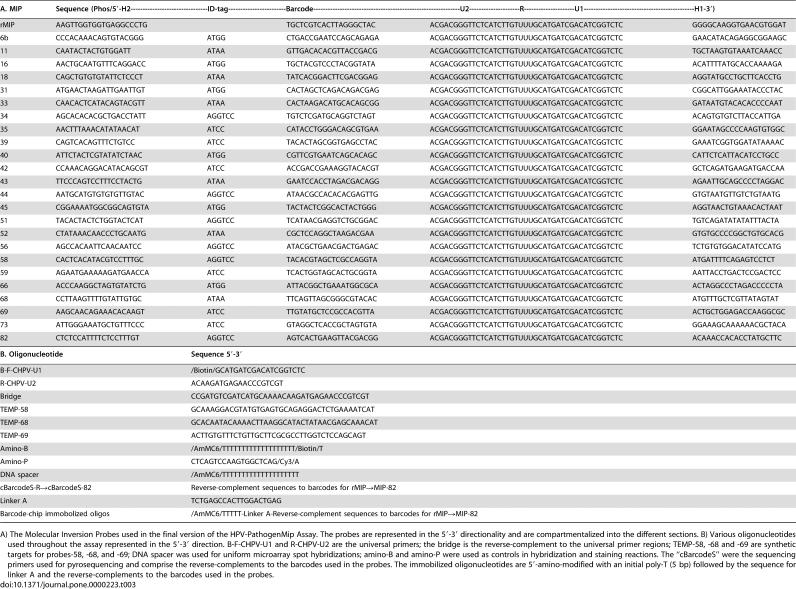
Oligonucleotides used in the HPV-PathogenMip Assay.

### Ligation-based probe construction

The full-length probes were designed to be of ∼110 base pairs. Each probe was synthetically manufactured in two shorter fragments, **A** and **B**, at about 50 base pair lengths, and with 5′-phosphorylated modifications (Table 3A). HPV-complementary portions of the probes were designed using PathogenMIPer, a previously described software tool for designing unique and specific probes, targeting any type of genome based on its sequence data in a FASTA file format [22]. The universal primer region was chosen for the point of the **A** and **B** fragment separation, 10 base pairs upstream (5′-3′ directional viewpoint) of the first dUTP residue. A complementary bridge oligonucleotide was designed (Table 3B) to hybridize to the universal primer region. Ligation was performed in a one-step reaction. Equal concentrations (2.5 µM) of construct **A**, construct **B**, and the bridge oligonucleotide were pooled together with 2.5 units of Ampligase (Epicenter Biotechnologies, Madison, WI), 5 units of AmpliTaq DNA Polymerase Stoffel Fragment (Applied Biosystems, Foster City, CA) and 1.25 mM of each nucleotide dCTP and dTTP (Fermentas, Hanover, MD). A GeneAmp PCR system 9700 (Applied Biosystems, Foster City, CA) thermocycler was used for initial heating at 95°C for 10 min, followed by 5 cycles of denaturation and hybridization/ligation at 95°C for 2 min and 50°C for 2 min. The ligation products were then run out on a Sub-Cell GT Agarose Electrophoresis System (Bio-Rad, Hercules, CA) using a 2.5% Ultra pure L.M.P Agarose gel (Invitrogen, Carlsbad, CA) with 5 µg/ml Ethidium Bromide (Sigma-Aldrich, St. Louis MO) staining. Gel bands of fragments at about a 100 bases were carefully cut out and dissolved in 0.4 M NaCl at 70°C. DNA extraction of the gel bands was based on a phenol and chloroform (Sigma-Aldrich, St. Louis, MO) organic extraction protocol. The aqueous phase was collected after organic phase separation with equal volumes of: i) phenol, ii) 1∶1 phenol/chloroform, and iii) chloroform. This procedure was followed by ethanol precipitation at −20°C over night. Glycogen (Sigma-Aldrich, St. Louis, MO) was added to facilitate DNA precipitation. The purified probes were normalized for probe concentration using a ND-100 Spectrophotometer (NanoDrop, Wilmington, DE).

### PathogenMip Assay

Prior to the assay, genomic DNA was fragmented by digestion with EcoRI and Xba I according to manufacturer's instructions (New England BioLabs; Ipswich, MA). MIP reactions were performed as follows: **i)** 200 ng of previously digested genomic DNA (or 50 ng undigested HPV plasmid DNA), 5 fmol each of 25 probes, 0.25 units of (Epicenter Biotechnologies, Madison, WI), 0.25 mM dGTP (Fermentas, Hanover, MD), and 1 units of AmpliTaq DNA Polymerase Stoffel Fragment (Applied Biosystems, Foster City, CA) in 1× Ampligase buffer (Epicenter Biotechnologies, Madison, WI) run in a total volume of 10 µl/rxn. After pre-incubation at 95°C for 10 min, the reaction went through 5 cycles of 95°C for 2 min, 50°C for 30 min and 37°C for 10 min. **ii)** 10 µl of 130 mM Tris-HCl (pH 7.8), 85.75 mM KCl, 3.85 mM MgCl_2_ and 0.1% BSA solution containing 5.6 units Exonuclease I and 112 units Exonuclease III (Epicenter Biotechnologies, Madison, WI) was added to each reaction. The mixture was incubated at 37°C for 60 min followed by enzyme deactivation at 80°C for 20 min. **iii)** 4 units of Uracil-DNA-Glycosylase (New England BioLabs, Ipswich, MA) was added to each reaction and incubated at 37°C for 20 min followed by enzyme deactivation at 80°C for 20 min. **iv)** PCR amplification was carried in a total reaction volume of 50 µl, containing 5 µl MIP reaction, GeneAmp 1X PCR Buffer II (Applied Biosystems, Foster City, CA), 2.5 mM MgCl_2_ (Applied Biosystems, Foster City, CA), 0.12 mM dNTPs (Fermentas, Hanover, MD), 2.5 units AmpliTaq Gold DNA polymerase (Applied Biosystems, Foster City, CA) and 0.2 µM of each universal primer B-F-CHPV-U1 and R-CHPV-U2 (Table 3B). A 10-min incubation step at 95°C was followed by 35 cycles of amplification with a thermocycler GeneAmp PCR system 9700 (Applied Biosystems, Foster City, CA). Each cycle included a denaturation step at 95°C for 45 sec, and an annealing step at 52°C for 30 sec. A final extension was done at 72°C for 5 min. For three randomly chosen reactions, an alternative PCR protocol was run in parallel, which was comprised of 60 cycles with an increased annealing temperature at 58°C. The latter protocol was used to test whether increased sensitivity of the assay could be achieved with a more extensive PCR amplification.

### Multiple-primer DNA pyrosequencing of barcodes

The B-F-CHPV-U1 primer used for universal probe amplification was biotinylated, and the leading strand could therefore be used as a sequencing template. Single strand template preparation was performed as previously described [23], and oligonucleotides complementary to barcodes included in the probes were used as sequencing primers (Table 3B). Each of the 24 HPV targeting probes contained one out of four ID-tags, which lay the basis for a rapid multiple-primer based screening by sequencing [5]. The primers were pooled into six mixes of four primers with different ID-tags (Table 2). Pyrosequencing was performed with a cyclic de novo sequencing dispensation (ACGT) using a PSQ^TM^HS96A DNA sequencing system.

### Microarray barcode-chip template preparation

For more efficient microarray hybridizations, single stranded DNA was generated from the first PCR amplification. Amplification was carried out in a total reaction volume of 50 µl, containing 1 µl from the first PCR reaction, 0.125× BD Titanium Taq DNA polymerase (Clontech, Mountain View, CA), 1X BD Titanium Taq PCR buffer (Clontech, Mountain View, CA), 0.2 mM dNTPs (Fermentas, Hanover, MD) and 0.2 µM of each universal primer B-F-CHPV-U1. A 10 min incubation step at 95°C was followed by 35 cycles of amplification with a thermocycler GeneAmp PCR system 9700 (Applied Biosystems, Foster City, CA). Each cycle included a denaturation step at 95°C for 45 sec, and annealing at 54°C for 30 sec. A final extension was performed at 68°C for 5 min.

### Barcode microarray preparation

Oligonucleotides were synthesized at the Stanford Genome Technology Center. Oligonucleotides used as probes on the array (Table 3B) consisted of (from 5′ to 3′): a 5′-amino group (for attachment to the array), a 5 bp poly-T sequence, Linker A, and a 20 bp complementary to barcode sequence. Probes were attached to the microarray essentially as described previously [24]. Each probe was printed in quadruplicate or quintuple, and two complete arrays were present on each chip. The post-printing processing of the microarrays was performed as recommended by the slide manufacturer (Amersham Biosciences, Piscataway, NJ). Control oligonucleotides used to verify array quality included a poly-T (20 bp) with 5′-amino and internal-biotin modification as a labeling control (amino-B), a oligonucleotide complementary to Linker A with internal-Cy3 (amino-P) as an internal control for each spot's quality, and a 5′-amino modified poly-T (20 bp) as a DNA spacer.

### Array hybridizations

In the hybridization step, the biotinylated single-stranded target and amino-P were applied to the microarray. For the initial target hybridization step, we used 50 µl of target DNA in 1× hybridization buffer (100 mM MES, 1 M [Na+], 20 mM EDTA, 0.01 Tween20), 1.25× Denhardt's solution, and 5 nM amino-P. The hybridization was performed at 42°C for 12–16 hours. After hybridization, the microarray was washed 3 times in wash buffer and then labeled for 10 min at 50°C with a solution containing streptavidin-allophycocyanin (1mg/ml final concentration), 6× SSPE, 1× Denhardt's solution, and 0.01% Tween-20. The microarray was scanned for fluorescence intensity at 535 nm and 635 nm using a GenePix 4000 fluorescent scanner (Axon Instrument, Foster City, CA) set to scan at 450 PMT. GenePix Pro software was used to determine the total fluorescence signal from each spot on the array.

### Validation and genotyping of DNA samples

For validation of the PathogenMip Assay, the 20 genomic DNA extracts were characterized for HPV presence and subtypes with nested PCR amplification using general primers PGMY09/11 and GP5+/6+ [1] according to a previously described protocol [26] with an initial amount of 40 ng/ml genomic DNA, followed by sequencing as described previously [5].

## References

[1] Gravitt PE, Peyton CL, Alessi TQ, Wheeler CM, Coutlee F (2000). Improved amplification of genital human papillomaviruses.. J Clin Microbiol.

[2] Csako G (2006). Present and future of rapid and/or high-throughput methods for nucleic acid testing.. Clin Chim Acta.

[3] Mackay IM, Arden KE, Nitsche A (2002). Real-time PCR in virology.. Nucleic Acids Res.

[4] Klaassen CH, Prinsen CF, de Valk HA, Horrevorts AM, Jeunink MA (2004). DNA microarray format for detection and subtyping of human papillomavirus.. J Clin Microbiol.

[5] Gharizadeh B, Zheng B, Akhras M, Ghaderi M, Jejelowo O (2006). Sentinel-base DNA genotyping using multiple sequencing primers for high-risk human papillomaviruses.. Mol Cell Probes.

[6] Chamberlain JS, Gibbs RA, Ranier JE, Nguyen PN, Caskey CT (1988). Deletion screening of the Duchenne muscular dystrophy locus via multiplex DNA amplification.. Nucleic Acids Res.

[7] Elnifro EM, Ashshi AM, Cooper RJ, Klapper PE (2000). Multiplex PCR: optimization and application in diagnostic virology.. Clin Microbiol Rev.

[8] Markoulatos P, Siafakas N, Moncany M (2002). Multiplex polymerase chain reaction: a practical approach.. J Clin Lab Anal.

[9] Bissonnette L, Bergeron MG (2006). Next revolution in the molecular theranostics of infectious diseases: microfabricated systems for personalized medicine.. Expert Rev Mol Diagn.

[10] Ji H, Kumm J, Zhang M, Farnam K, Salari K (2006). Molecular inversion probe analysis of gene copy alterations reveals distinct categories of colorectal carcinoma.. Cancer Res.

[11] Landegren U, Kaiser R, Sanders J, Hood L (1988). A ligase-mediated gene detection technique.. Science.

[12] Landegren U, Kaiser R, Caskey CT, Hood L (1988). DNA diagnostics–molecular techniques and automation.. Science.

[13] Nilsson M, Malmgren H, Samiotaki M, Kwiatkowski M, Chowdhary BP (1994). Padlock probes: circularizing oligonucleotides for localized DNA detection.. Science.

[14] Nilsson M, Krejci K, Koch J, Kwiatkowski M, Gustavsson P (1997). Padlock probes reveal single-nucleotide differences, parent of origin and in situ distribution of centromeric sequences in human chromosomes 13 and 21.. Nat Genet.

[15] Gustafsdottir SM, Nordengrahn A, Fredriksson S, Wallgren P, Rivera E (2006). Detection of individual microbial pathogens by proximity ligation.. Clin Chem.

[16] Szemes M, Bonants P, de Weerdt M, Baner J, Landegren U (2005). Diagnostic application of padlock probes–multiplex detection of plant pathogens using universal microarrays.. Nucleic Acids Res.

[17] Hardenbol P, Baner J, Jain M, Nilsson M, Namsaraev EA (2003). Multiplexed genotyping with sequence-tagged molecular inversion probes.. Nat Biotechnol.

[18] Hardenbol P, Yu F, Belmont J, Mackenzie J, Bruckner C (2005). Highly multiplexed molecular inversion probe genotyping: over 10,000 targeted SNPs genotyped in a single tube assay.. Genome Res.

[19] Wang Y, Moorhead M, Karlin-Neumann G, Falkowski M, Chen C (2005). Allele quantification using molecular inversion probes (MIP).. Nucleic Acids Res.

[20] de Villiers EM, Fauquet C, Broker TR, Bernard HU, zur Hausen H (2004). Classification of papillomaviruses.. Virology.

[21] Doorbar J (2006). Molecular biology of human papillomavirus infection and cervical cancer.. Clin Sci (Lond).

[22] Thiyagarajan S, Karhanek M, Akhras M, Davis RW, Pourmand N (2006). PathogenMIPer : A tool for the design of molecular inversion probes to detect multiple pathogens.. BMC Bioinformatics.

[23] Gharizadeh B, Akhras M, Nourizad N, Ghaderi M, Yasuda K (2006). Methodological improvements of pyrosequencing technology.. J Biotechnol.

[24] Dufva M (2005). Fabrication of high quality microarrays.. Biomol Eng.

[25] Gharizadeh B, Akhras M, Unemo M, Wretlind B, Nyren P (2005). Detection of gyrA mutations associated with ciprofloxacin resistance in Neisseria gonorrhoeae by rapid and reliable pre-programmed short DNA sequencing.. Int J Antimicrob Agents.

[26] Fuessel Haws AL, He Q, Rady PL, Zhang L, Grady J (2004). Nested PCR with the PGMY09/11 and GP5(+)/6(+) primer sets improves detection of HPV DNA in cervical samples.. J Virol Methods.

[27] Eason RG, Pourmand N, Tongprasit W, Herman ZS, Anthony K (2004). Characterization of synthetic DNA bar codes in Saccharomyces cerevisiae gene-deletion strains.. Proc Natl Acad Sci U S A.

[28] Landegren U (1993). Ligation-based DNA diagnostics.. Bioessays.

[29] Nielsen CB, Singh SK, Wengel J, Jacobsen JP (1999). The solution structure of a locked nucleic acid (LNA) hybridized to DNA.. J Biomol Struct Dyn.

[30] Pierce SE, Fung EL, Jaramillo DF, Chu AM, Davis RW (2006). A unique and universal molecular barcode array.. Nat Methods.

[31] Pettersson E, Lindskog M, Lundeberg J, Ahmadian A (2006). Tri-nucleotide threading for parallel amplification of minute amounts of genomic DNA.. Nucleic Acids Res.

[32] Fire A, Xu SQ (1995). Rolling replication of short DNA circles.. Proc Natl Acad Sci U S A.

[33] Holmberg A, Blomstergren A, Nord O, Lukacs M, Lundeberg J (2005). The biotin-streptavidin interaction can be reversibly broken using water at elevated temperatures.. Electrophoresis.

[34] Latorra D, Arar K, Hurley JM (2003). Design considerations and effects of LNA in PCR primers.. Mol Cell Probes.

[35] Schaeffer AJ, Nguyen M, Liem A, Lee D, Montagna C (2004). E6 and E7 oncoproteins induce distinct patterns of chromosomal aneuploidy in skin tumors from transgenic mice.. Cancer Res.

